# Expansion Characteristics and Shear Behavior of Reinforced Concrete Beams Under Non-Uniform Expansion Induced by Alkali–Silica Reaction

**DOI:** 10.3390/ma18020312

**Published:** 2025-01-11

**Authors:** Feng Sheng, Xuehui An, Mengliang Li, Yuxiang Zhou, Pengfei Li

**Affiliations:** 1 State Key Laboratory of Hydroscience and Engineering, Tsinghua University, Beijing 100084, China; feng_shengpcr@163.com; 2 PowerChina Roadbridge Group Co., Ltd., Beijing 100048, China; menglianglipcrg@163.com (M.L.); yuxiangzhoupcrg@163.com (Y.Z.); 3 Engineering Research Centre of Diagnosis Technology of Hydro-Construction, Chongqing Jiaotong University, Chongqing 400074, China; lipengfei@cqjtu.edu.cn

**Keywords:** alkali–silica reaction, reinforced concrete structure, restraint effect, non-uniform damage, deterioration mechanism

## Abstract

Alkali–silica reaction (ASR) is an important factor that seriously affects the durability of reinforced concrete (RC) structures. The current research on alkali-aggregate mainly focuses on the deterioration mechanism of materials and the mechanical properties of standard specimens. However, there is a gap in the field of research on the effect of alkali-aggregate damage on the level of RC structures. In this study, five RC beams were tested, and the depth and location of alkali solution immersion were used as the test variables, with the aim of investigating how the steel reinforcement suppresses the expansion caused by ASR and evaluating the shear behavior of RC beams after non-uniform ASR damage. The results of the study showed that immersion in an alkali solution and an increase in immersion depth accelerated the rate of expansion development, while steel reinforcement inhibited the rate of expansion development. Compared with undamaged RC beams, ASR initially generates expansion stresses within the concrete, which increase the cracking and yield loads of RC beams and delay the cracking of RC beams, and ASR reduces the ultimate load-carrying capacity and ductility of RC beams due to the disruption of the concrete microstructure. Finally, a chemo-mechanical analysis method is proposed based on experimental results, which incorporate an ASR expansion model and a pore mechanics model. The efficacy and precision of this model are validated through comparison with experimental results.

## 1. Introduction

Alkali–silica reaction (ASR) is an internal deleterious phenomenon in concrete, resulting from the interaction between reactive silica in the aggregate and alkalis within the pore solution, leading to gel formation [1,2,3]. Owing to its hydrophilic nature, the gel is capable of absorbing water from its surroundings, which leads to expansion and subsequently causes material cracking and deterioration of mechanical properties [4]. Extensive research investigations have demonstrated that ASR induces degradation in the mechanical properties of concrete, including the Poisson ratio, elastic modulus, and tensile strength [5,6,7,8].

ASR is commonly found in the concrete of infrastructure structures such as bridges, dams, and tunnels. At the RC structural level, ASR-induced concrete expansion and cracking can be mitigated by internal reinforcement restrictions or external boundary conditions. Several experiments [9,10,11] have been conducted to investigate the mechanical properties of ASR-damaged concrete under stress confinement. Fiset et al. [12] conducted a series of experiments to investigate the influence of ASR-distressed RC on aggregate interlock, revealing that chemical prestressing yields beneficial effects, while ASR-induced damage adversely affects aggregate interlock in affected specimens. Consistent with their previous research [13], pullout tests were conducted on specimens exhibiting varying levels of ASR expansion to quantitatively assess its impact on bond performance in reinforced concrete, revealing that ASR can enhance bond performance at relatively low volumetric expansion rates. The size effect of ASR products has been emphasized [14], and caution was also advised when extrapolating results from standard specimens to structures. Consequently, numerous test programs have been conducted to investigate the structural performance of ASR-damaged RC structures. The experimental findings [15,16,17] indicate that beams affected by ASR damage demonstrate comparable flexural performance to undamaged beams. Ahmed et al. [18] reported a marginal increase in flexural strength for damaged beams based on experimental evidence. However, the impact of ASR damage on shear strength in RC structures remains underexplored in previous studies. Thus, this study focuses specifically on the shear behavior of RC beams subjected to ASR damage. Furthermore, in the case of large civil engineering infrastructures, such as concrete dams, where loading directions and reinforcement alignments result in anisotropic stress states [19], it is also necessary to consider the impacts of stresses and constraints that arise from reinforcement or boundary conditions during ASR.

Numerous models have been developed at both the microscopic [20] and macroscopic scales [21] to predict the ASR process and its impact on mechanical behavior. In order to anticipate the mechanical properties of ASR-damaged RC structures and evaluate their structural safety, researchers have progressively shifted their research focus toward the macroscopic modeling of ASR. The Charlwood model [22] has been widely used to analyze the expansion deformation caused by alkali-aggregate reaction (AAR) in concrete structures, including expansion deformation caused by ASR and alkali carbonate reaction (ACR). Tanaka et al. [23] conducted a structural-level simulation to investigate the mechanical properties of slabs damaged by ASR. Larive [24] summarized the expansion deformation pattern of a significant number of concrete prism specimens that were subjected to accelerated AAR tests for 400 days at 38 °C, and it was found that the AAR expansion deformation of concrete follows an S-shaped curve.

In recent years, there has been a growing interest in chemo-mechanical models that integrate the principles of ASR expansion with concrete mechanics [21,25]. Based on the AAR expansion deformation curve proposed by Larive, a thermo-chemo-mechanical model was developed by Ulm et al. [21]. This model can be employed to investigate the evolution of stress–strain in gravity dams under the combined effects of temperature, ASR, and stress. Subsequently, this model has been further refined and developed to study the impact of ASR on concrete structures. The dual-porosity damage model based on the Larive model, introduced by Comi et al. [25], is developed from fracture energy principles and is capable of simultaneously describing the interaction between concrete expansion and cracking. These findings have been successfully applied to studying the concrete damage characteristics of the Fantana Dam. Here, the model not only considers the mechanisms of ASRs but also takes into account the mechanical damage of concrete materials, accurately simulating the mechanical behavior of concrete under the influence of ASR. However, these studies have overlooked the non-uniformity of ASR expansion in RC structures and have not considered the impact of boundary conditions, such as reinforcement, on ASR expansion. Previously, the authors [26] proposed a chemo-mechanical analysis method to calculate ASR expansion and bond properties while considering the restraining effect of reinforcement bars. The accuracy of this method was validated by comparing the predicted results with experimental data. However, the complexity of the model lies in establishing reinforced simulation units to mimic the constraints between steel and concrete. This complexity makes the method impractical for large-scale RC structures and necessitates further research.

The objective of this paper is to investigate the shear behavior of an RC beam under inhomogeneous expansion caused by ASR. Firstly, accelerated ASR tests were conducted on beam specimens immersed at various depths and positions in an alkali solution to measure expansion. Subsequently, three-point bending shear tests were carried out on RC beams to investigate the effects of ASR damage on the shear behavior of RC structures.

## 2. Experimental Procedures

### 2.1. Materials

The cement used in the experiment was an ordinary silicate cement (PO 42.5 produced by Red Lion Holding Group Co., Zhejiang, China), which complied with the national standard [27]. The chemical composition of the cement is shown in Table 1, while the concrete mix proportions are presented in Table 2. The water–cement ratio was 0.45, the particle size of coarse aggregate ranged from 5 to 20 mm, and the fineness modulus of fine aggregate was 2.4. The reactivity of both fine and coarse aggregates was evaluated through an accelerated mortar bar test. According to the test code [28], the expansion behavior of mortar bars in an alkaline solution at 80 °C was investigated. The results showed that in the fine aggregate mortar bar test, the average 14-day expansion of the three sample mortar bars was 0.30%, with a coefficient of variation of 13.55%. In the coarse aggregate mortar bar test, the average expansion of the three sample mortar bars after 14 days was 0.23%, with a coefficient of variation of 7.46%. The results indicated that both the fine and coarse aggregate were reactive, as the expansion of the mortar bars exceeded the established limit (0.10%) after 14 days of immersion. It is necessary to note that the type of water-reducing agent chemistry used in this study is a polycarboxylic acid high-efficiency water-reducing agent, which does not result in an increase in ASR expansion of concrete [29,30].

### 2.2. Test Specimens

**Figure 1:**
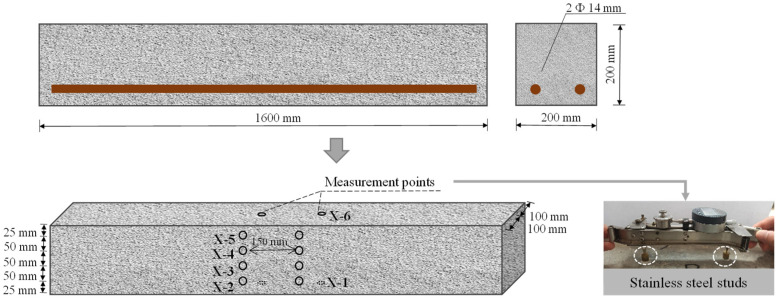
Arrangement of beams and locations for measurements.

The beam-casting design is based on experiments conducted by Hayashida [31]. The literature mentions that the dimensions of the beam are 200 mm × 200 mm × 1600 mm, with a cover thickness of 50 mm. In this study, five RC beams were cast with the same size and rebar configuration. Figure 1 illustrates details of the beam specimens, which have dimensions of 200 mm × 200 mm × 1600 mm. Two deformed reinforcing bars (with an average measured yield strength, *f_y_*, of 521 MPa) with a diameter of 14 mm were arranged as the longitudinal steel bars at the bottom of the beams. The concrete cover for the longitudinal rebar was set to be 50 mm.

As shown in Figure 1, each specimen was equipped with six measuring points to monitor expansion during the test. In the vertical direction, measurements were taken at specific depths: 0 mm (X-1), 25 mm (X-2), 75 mm (X-3), 125 mm (X-4), 175 mm (X-5), and 200 mm (X-6). In this context, “X” refers to the beam specimens, and the number signifies the measurement depth. Custom-made stainless-steel studs were attached to each specimen, maintaining an initial approximate distance of 150 mm between each measuring point. Considering the structural stress characteristics of the beam, including tension and compression areas, as well as the extent of expansion changes, measurement points are evenly distributed at symmetrical positions within the structure. In this study, only the confining effect of reinforcement is taken into account for the impact of boundary conditions on expansion rate. Therefore, no measurement points were placed at the edges of the beam.

### 2.3. ASR Development

After 28 days of curing, all specimens were placed in tanks containing a 2.8 mol/L NaOH solution to accelerate ASR. Despite the fact that an increase in alkali content leads to increased ASR swelling and cracking [32], this accelerated condition has been utilized in several research studies [33,34]. In this study, based on the literature mentioned above, the concentration of the immersed NaOH solution was set at 2.8 mol/L. In actual engineering environments, the occurrence of ASR is extremely slow. The rate of ASR is correlated with the characteristics of concrete raw materials, the alkali content, and environmental parameters such as temperature and humidity. To accelerate the expansion associated with ASR, this experiment utilized a relatively high concentration of NaOH solution. And the annual average temperature of the NaOH solution is 20 °C, and the temperature of the NaOH solution varies with the atmospheric temperature. During the experiment, the variation in the NaOH solution level within the tanks was closely monitored. Regular acid-base titration tests were conducted, and the NaOH solution was replenished as needed. To ensure that the N-beam undergoes reinforcement yielding failure mode, thereby avoiding brittle failure or excessive deformation that may occur with over-reinforced or under-reinforced beams, the design reinforcement ratio is set at 0.77%. This approach aims to study the changes in failure modes of RC beams after experiencing non-uniform alkali-aggregate expansion damage. The specific design parameters of the experimental beam are detailed in Table 3.

**Figure 2:**
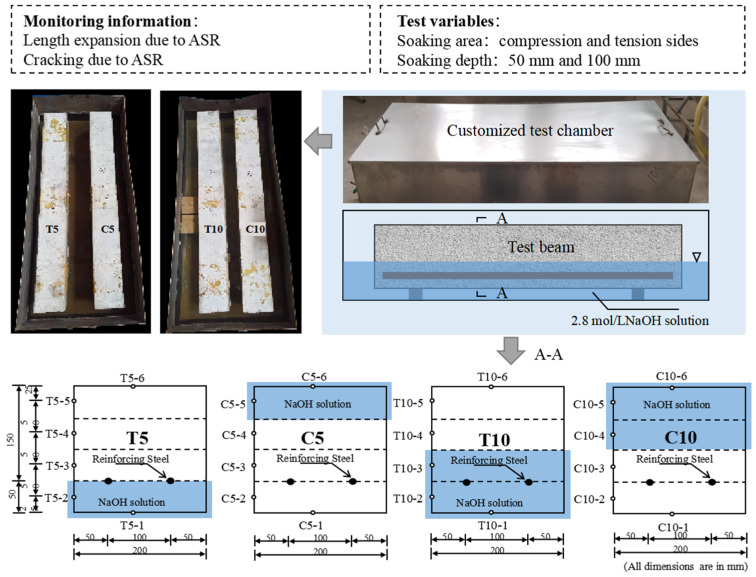
Schematic of the immersion process in NaOH solution.

To investigate the impact of environmental factors on material performance, this study designed experiments considering two primary experimental variables: the area of solution immersion (tensile and compressive zones) and the depth of solution immersion (50 mm and 100 mm), as illustrated in Figure 2. Here, ’C’ and ’T’ represent the deterioration on the compression and tension sides, respectively. The specimen that has not undergone an accelerated alkali–silica reaction (ASR) test is denoted as ’N’. Before being placed into the test tanks, the initial distance between measurement points (*l*_0_) was determined using a handheld strain gauge with an accuracy of 0.0001 mm. Throughout the 34-month duration of the experiment, the distance between the measurement points (*l_t_*) was recorded monthly. The expansion rate was then calculated at each measurement point according to Equation (1). Additionally, surface cracks on the specimen were regularly inspected with the assistance of a magnifying glass, and specific areas were selected for detailed crack mapping.

(1) $$\varepsilon_{t}=\frac{l_{t}-l_{0}}{l_{0}}\times 100\%$$

where *ε_t_* is the expansion rate of the specimen in month *t*, *l*_0_ is the initial distance between measurement points, and *l_t_* is the distance between measurement points at *t* month.

In this study, the non-uniform accelerated expansion of RC beams was investigated, and the following mechanisms were identified. After immersing RC beams in alkali solutions at varying depths, different sections of the beams exhibited varying expansion rates in the vertical direction due to the restraining effect of the reinforcement. This phenomenon is caused by the conduction transport of the alkali solution within the pores of the RC beam. The restraining effect of the reinforcement leads to varying expansion responses of the alkali-aggregate in different parts of the RC beams. In this study, specific immersion depths and NaOH solution concentrations were employed for accelerated ASR testing. Other factors influencing the ASR, such as the amount of fly ash incorporated, curing time, and water-to-cement ratio [35], will be thoroughly discussed in subsequent research.

### 2.4. Experimental Investigation of Mechanical Properties

**Figure 3:**
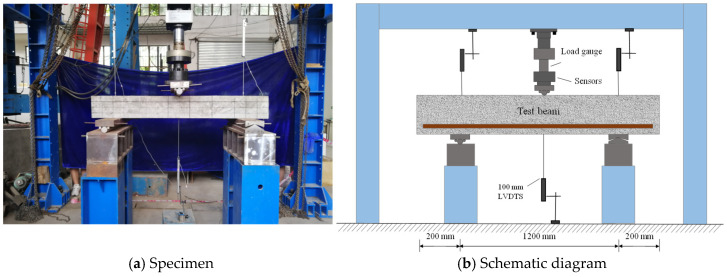
The setup for shear loading tests.

After 34 months of accelerated ASR testing, according to the China code GB/T 50152 [36], shear tests were carried out to investigate the shear behavior of RC beams with ASR deterioration. The setup of the loading test is shown in Figure 3. During the experiment, a simply supported specimen was subjected to a load applied at mid-span. Three linear variable differential transducers (LVDTs) with a measurement range of 0–100 mm were positioned at the two supports and mid-span for displacement measurements. The employed test graded loading, where each step corresponded to approximately 10% of the ultimate load while reducing the load to 5% of the expected ultimate load as it approached both shear cracking and ultimate loads. The test concluded with concrete crushing occurring at the top surface of the compression zone.

## 3. Expansion and Cracking Due to ASR

### 3.1. The Expansion Caused by ASR

**Figure 4:**
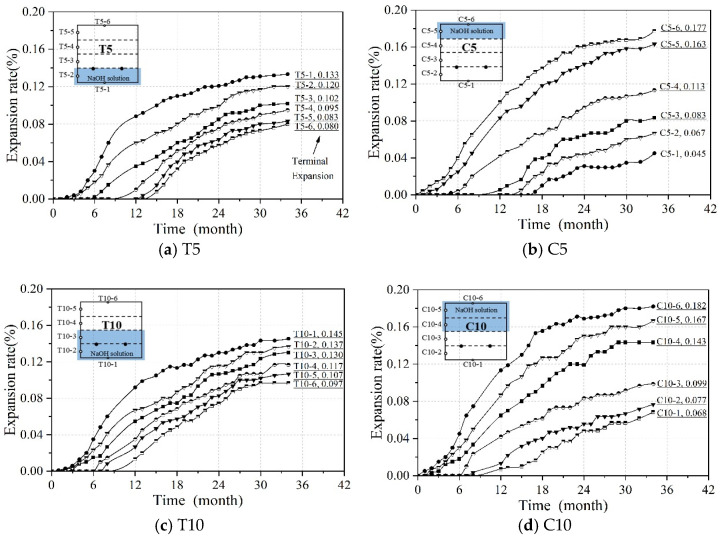
Length expansion of beams: (**a**) T5 beam, (**b**) C5 beam, (**c**) T10 beam, and (**d**) C10 beam.

Figure 4 illustrates the change in expansion rate for each measurement point on the beam. The expansion rate of each beam gradually increases with the duration of monitoring. From 0 to 6 months, ASR shows a slow increase in expansion rate, and there is no expansion at some measured points. The expansion rate increases rapidly from 6 to 24 months. Afterward, it starts decelerating and tends to stabilize between 24 and 34 months. This observation is consistent with the curve of Larive [24], which was derived from a comprehensive analysis of numerous experiments. The development of ASR can be divided into three stages: initiation, development, and plateau periods. During the initiation stage, the ASR gel initially fills existing pores in concrete without causing macroscopic expansion. In this study, the initiation phase is defined as the first 6 months of ASR. During the subsequent development stage, as more gel is produced and initial pores are filled, the pressure exerted by the gel on pore walls leads to an increase in expansion magnitude. The developmental period in this study ranged from the 6th to the 24th month of the ASR. Finally, when reactants are depleted, and no additional gel is supplied, pressure remains constant and macroscopic expansion stops completely. The plateau periods in this experiment occurred between months 24 to 34 of the ASR. Additionally, the shrinkage phenomenon observed in the concrete beams during ASR expansion monitoring will be further investigated in future studies.

However, it should be noted that unlike most previous studies which have suggested uniformity in ASR-induced expansions, the findings in this study demonstrate non-uniformity, both in terms of development rates and maximum values across different measurement points. As shown in Figure 4a,c, although the tension zone of the reinforced concrete beam is immersed in NaOH solution, the compression zone is also affected by ASR, leading to expansion. This phenomenon occurs due to the transport of the alkaline solution along the height of the reinforced concrete beam cross-section. The alkali solution migrates through the pores in the beam, from the tension zone to the compression zone, where the expansion reaction takes place. Moreover, during the immersion of the tension zone, the onset of expansion in the compression zone occurs later than in the tension zone. This delay is attributed to the relatively slow rate of transport and expansion reactions of the alkali solution within the reinforced concrete beam.

Comparing Figure 4a,b, it can be observed that, under the same immersion depth, the expansion values at measuring points 1 and 2 in the tension zone (T5) are consistently lower than those at measuring points 5 and 6 in the compression zone (C5) across all time intervals. Additionally, the final expansion rate at point C5-6 is 33.08% higher than that at T5-1. This is attributed to the restraining effect of the reinforcement in the tension zone, which reduces the ASR-induced expansion in that region. In contrast, during immersion of the compression zone, the absence of reinforcement allows the ASR to occur more freely within the compression zone concrete. A similar phenomenon is observed when comparing Figure 4c,d.

Furthermore, as shown in Figure 4, the partial immersion of the reinforced concrete beam in NaOH solution leads to a non-uniform distribution of the expansion rate along the height of the beam cross-section. Throughout the different time intervals, the expansion rates at the measuring points fully immersed in the alkaline solution are consistently higher than those at the points not immersed in the solution. For instance, the final expansion rate at point T5-1 is 66.25% higher than that at point T5-6. This phenomenon is primarily attributed to the slow transport of the alkaline solution within the reinforced concrete beam. The regions immersed in the alkaline solution have a higher availability of water and alkaline ions, such as OH^−^, which participate more actively in the ASR, resulting in a higher expansion rate. In contrast, the regions not exposed to the alkaline solution have lower water and alkali content, leading to a lower expansion rate.

### 3.2. Impact of the Immersion Position

**Figure 5:**
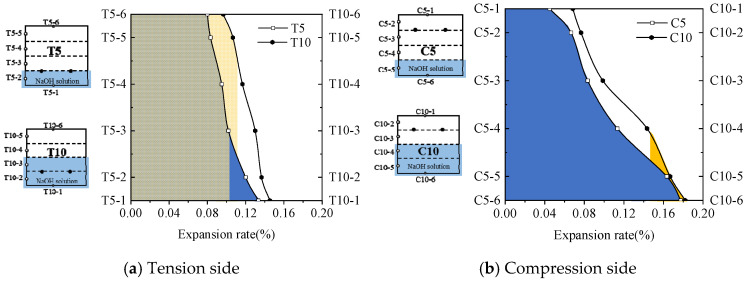
Impact of immersion position.

Figure 5 shows the distribution of expansion rates at month 34. The expansion rate of the measurement points is observed to be higher when directly immersed in the solution, while it decreases as the distance from the solution increases. For T5, the measurement point T5-1 located in the solution exhibited a final swelling rate of 0.133%, whereas T5-6, situated farthest from the solution, had a final swelling rate of 0.080%, resulting in a difference of 0.053%. Similar trends were observed for C5, T10, and C10, with maximum-minimum differences in expansion rate on beams of 0.132%, 0.048%, and 0.114%, respectively. These findings are consistent with the observations [37] that partial immersion creates moisture gradients vertically, leading to non-uniform swelling in this direction. In this study, accelerated ASR testing was conducted through partial immersion in a sodium hydroxide solution, where reactants such as water, OH-, and Na+ ions were transported upward via capillary action [38]. The lower reactants became more abundant while the upper ones gradually decreased, causing non-uniform swelling.

## 4. Beam Shear Loading Tests

### 4.1. Effect of ASR on Structural Performance of RC Beams

#### 4.1.1. The Impact of ASR Damage on the Cracking Load

The results presented in Table 4 indicate that the cracking loads of the RC beams increase after experiencing ASR damage compared to the undamaged state (Beam N). Beams C5 and C10, where the immersed area is in the compression zone, show a smaller increase in cracking load. In contrast, beams T5 and T10, where the immersed area is the tension zone, exhibit a significant increase in cracking load. Among them, T10, with a greater immersed depth, shows the most substantial increase. Specifically, beam T10 exhibits a 26.1% increase in cracking load compared to beam N, and beam C10 shows an elevated cracking load of 8.7%. These findings suggest that ASR induces higher levels of cracking on RC beams, particularly when the soaked area is located within a tensile zone. The mechanical properties of the two types of beams were compared by conducting tests on active and inactive beams, revealing an increase in the cracking load of RC beams after ASR deterioration [17]. The longitudinal steel bars in reinforced beams effectively confine the expansion caused by ASR, inducing tension in the steel bars and compression in the surrounding concrete. Consequently, the compressive stress developed in the concrete can be regarded as a form of chemical pre-stress. During the test, the concrete was required to withstand this stress without cracking, thus increasing the cracking load. As discussed earlier, when the immersion zone experiences tensile forces, it induces higher expansion rates in the surrounding concrete and generates greater pre-compressive stresses due to reinforcement confinement. Consequently, there is a more pronounced enhancement in the cracking load.

#### 4.1.2. Effects of ASR Damage on the Maximum Load

**Figure 6:**
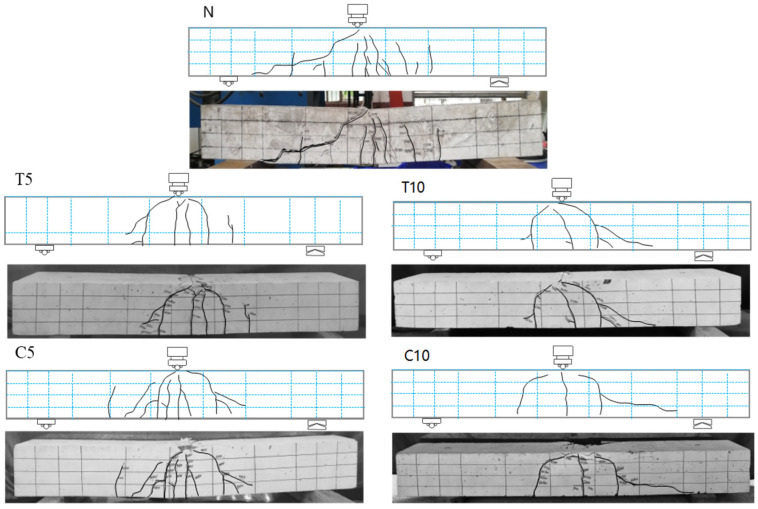
Failure mode of test specimens.

Table 5 shows that the ultimate load decreases with increasing depth of immersion in the alkali solution, with a 5.6% and 10.9% decrease in ultimate load capacity for T10 and C10, respectively, compared to beam N. This indicates that ASR reduces the shear load-carrying capacity of RC beams, and for beams where the immersed region is in compression, the decrease in shear load capacity caused by ASR is more pronounced. Aryan et al. [5] also validated through shear tests on beams, that compared to beams with an ASR expansion rate of 0.2%, beams with a 0.4% expansion rate exhibited a 6% decrease in shear strength and a 25% reduction in shear stiffness. Additionally, the former showed approximately twice as many shear cracks and shear deformations at the peak load. Saouma et al. [39] conducted a research study consisting of 648 analyses, which revealed that 53% of the analysis results indicated a decrease in overall shear strength after ASR, while 47% showed an increase in overall shear strength. At the material level, ASR leads to micro and macro cracks, which reduce the tensile strength of concrete and affect its shear strength. However, at the structural level, the expansion of reinforcing steel provides confinement for concrete, enhancing its shear strength. Comparing the findings of this study with those of the aforementioned researchers reveals that ASR reduces the shear capacity of RC beams, particularly when the compression zone is affected by ASR damage, resulting in significant deterioration in shear capacity. In this test, the reduction in shear strength caused by the concrete played a major role due to the lack of hoop configuration. As depicted in Figure 6, the beam immersed in the compression zone exhibited many cracks in this region. The ASR response resulted in substantial damage to the compressive strength of concrete, thereby leading to a more pronounced reduction in maximum load.

#### 4.1.3. Effects of ASR Damage on the Deformation Behavior

The ductility of a structural element refers to its capacity to undergo plastic deformations prior to failure while maintaining a significant portion of its strength. A ductile system exhibits ample warning signs before experiencing catastrophic failure. Ductility factors are typically quantified by various deformation-related parameters, including displacement, curvature, and energy. In this study, the displacement ductility index is utilized to characterize the ductility of each beam. The displacement ductility index *μ* is calculated using Equation (2), where the ultimate deflection ($\Delta_{u}$) and tension steel yield deflection ($\Delta_{y}$) are defined as parameters in the load-deflection curves.

(2) $$\mu =\Delta_{u}/\Delta_{y}$$

Table 6 shows that ASR degradation causes a reduction in RC beam ductility and that ductility decreases with increasing depth of solution immersion. When the immersion depth is 10 cm, the ductility index of beams with the immersion area being the tensile and compressive zones decreases by 54% and 44%, respectively. This is consistent with the results of the experiment [40]. This is because ASR generates hygroscopic ASR gel, which causes the concrete to expand and increase in volume. On one hand, this volumetric expansion leads to the formation of numerous microcracks and capillary cracks within the concrete, which weaken the bond between the aggregates and the cement paste, thereby affecting the overall strength and toughness of the concrete. On the other hand, under loading, the microcracks and capillary cracks induced by the ASR further reduce the overall toughness of the concrete and impair the bond between the concrete and the reinforcement.

### 4.2. Failure Modes of the Specimen

Table 7 shows the results of the static loading tests, where *P_cr_* is the cracking load, *P_y_* is the yield load, and *P_u_* is the ultimate load. Compared to the N group, the beams affected by ASR exhibited a higher cracking load and yield load. This can be attributed to the interaction between the steel reinforcement and the concrete through bond forces, which provide confinement during the expansion process. The confinement effect prevents the concrete from freely expanding, leading to an increase in internal stresses within the concrete. The accumulation of these internal stresses makes it more difficult for the concrete to crack and yield under external loading [19,41]. The ratio of *P_cr_* to *P_u_* tends to increase with increasing depth of deterioration in the tension zone for RC beams as compared to N beams. However, except for T5, where the ultimate load is slightly increased, all other beams have a reduced ultimate load after being affected by ASR. Furthermore, all beams exhibit shear compression failure after flexural yield [42].

The failure mode of test specimens is illustrated in Figure 6. During the loading process, initial vertical cracks appear in the tensile zone, followed by critical diagonal cracks when a certain load strength is reached. These critical diagonal cracks propagate towards\ the mid-span loading point, maintaining an intact compressed concrete section. The concrete remained intact until the region near the top of the diagonal crack was crushed due to the combined effect of shear and compressive stresses, ultimately leading to the shear-compression failure of the beam after it reached flexural yield. The damage process occurs gradually, with higher loads required for failure compared to when diagonal cracks first appear. Additionally, there appears to be a decreasing trend in crack formation after exposure to ASR, and an increase in immersion depth of the solution results in fewer cracks. For the undamaged beam N, there were initially eight cracks. However, beams with a solution immersion depth of 10 cm (C10 and T10) exhibited only three cracks, representing a reduction of 62.5%. Furthermore, ASR-affected beams showed an increased average crack spacing. Since RC beams experiencing ASRs may rapidly form larger cracks in localized areas and penetrate under shear, rather than producing a larger number of smaller cracks, resulting in a reduction in the number of cracks.

### 4.3. Load-Deflection Curves

**Figure 7:**
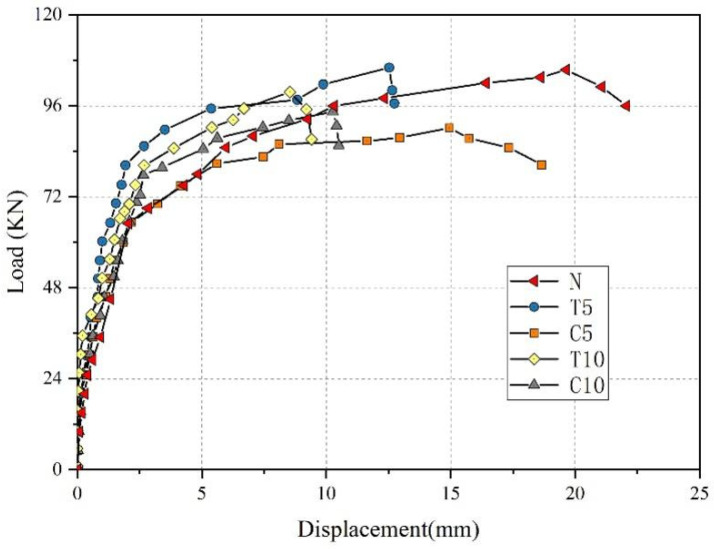
Load-deflection curves.

Figure 7 shows the load-deflection curve for each beam. Before cracking, the load-deflection curves of the test beams largely overlap, and ASR increases the cracking load. The slope of the load-deflection curve of the test beam decreases after cracking until the reinforcement yields. However, specimens subjected to ASR exhibit smaller displacements for the same load. When longitudinal bars yield, the load increases slowly, the deflection rapidly increases, and the stiffness of the test specimen rapidly decreases. Eventually, concrete in the compression zone cracked, and shear failure occurred in all RC beams. However, there were significant differences in the ultimate loads and final deflections among the beams. The experimental results on the impact of ASR damage on the shear performance of reinforced concrete beams indicate that, in practical engineering applications, the prevention of ASR should focus on minimizing the occurrence of ASRs in the compression zone of reinforced concrete beams. This is because the ultimate load-carrying capacity of the beam is at its lowest when ASR occurs in the compression zone. For reinforced concrete beams subjected to ASR in the tensile zone, incorporating fiber materials into the beam can enhance the structure’s maximum deflection.

## 5. Coupled Chemo-Mechanical Analysis Methods

### 5.1. Basic Model of Coupled Chemo-Mechanical Analysis Methods

This study uses the multi-scale simulation platform of the DuCOM-COM3, a multiscale chemo-hygro computational system [43], as the platform for analytical research. Specifically, the DuCOM calculation for concrete materials is used to obtain parameters such as strength, modulus of elasticity, drying shrinkage, and strain. These data are then transferred to the COM3 mechanical load calculation system, which performs mechanical analysis calculations based on the input conditions, taking into account the drying shrinkage strain transferred from DuCOM and the strength, modulus of elasticity, and other parameters to calculate the reinforcement strain and crack distribution information of the reinforced concrete structure. The method of chemo-mechanical analysis used is similar to that previously used by the authors [26,39,40,44,45,46,47,48]. The method establishes a skeleton element, where the pore element calculates the expansion stress of ASR products during chemical reactions, while the skeleton element calculates the mechanical state of concrete structure in terms of expansion damage. Subsequently, by employing a pore mechanics model, this study achieves coupling analysis between microscopic pore expansion and fine skeleton stress damage in concrete structures, thereby reproducing the mechanical property damage caused by ASR.

**Figure 8:**
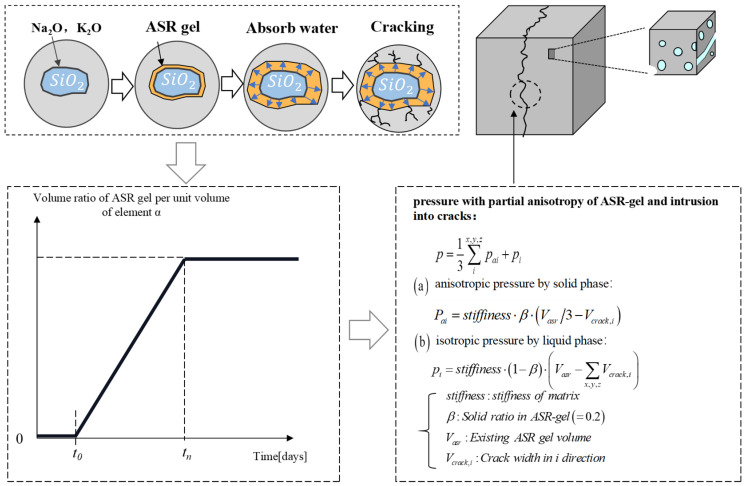
Scheme for calculating ASR-gel formation and stress information. [50].

Takahashi et al. [49] developed an ASR swelling model to simulate the generation and migration of ASR gels. In the model, ASR gel is considered semi-liquid. The total pore pressure *p* can be obtained by summing the anisotropic pressure *p_ai_* in the solid fraction and the isotropic pressure *p_i_* in the liquid fraction. In this study, based on the model proposed [50], a simplified ASR process was adopted. This method does not consider the chemical process of ASR or the formation and migration of ASR gel. The generation of ASR gel is represented using a simplified linear function, where the volume ratio of ASR gel per element volume is denoted as *α*. The start time of ASR, *t*_0_, and the end time, *t_n_*, are key parameters for calculating the generation process of ASR gel, as shown in Figure 8.

**Figure 9:**
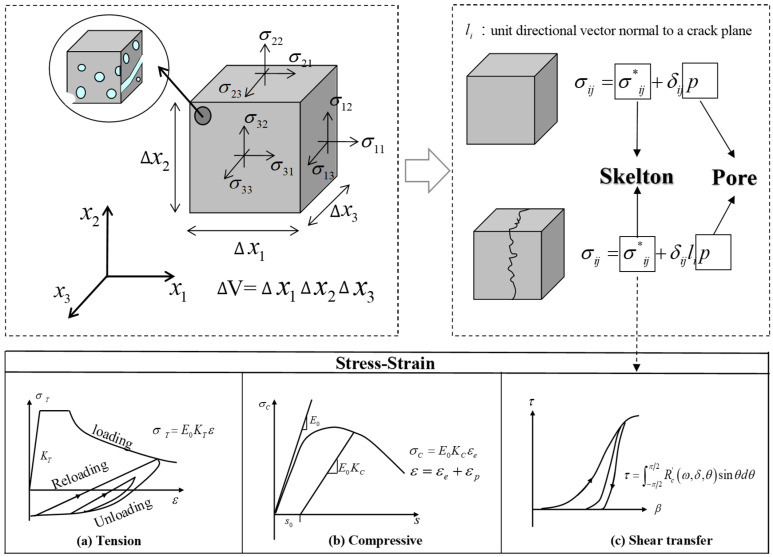
Foundational concept of the poro-mechanical model [50].

As shown in Figure 9, to convert microscopic pore pressure into macroscopic stress in concrete, this study adopts a pore mechanics model based on Biot’s theory [51]. ASR gel is considered as the medium within the concrete skeleton pores and cracks. Prior to cracking, the total stress *σ_ij_* can be obtained by simply summing up the skeleton stress σ^*^_ij_ and pore pressure *p*, as shown in Equation (3).

(3) $$\sigma_{ij}=\sigma_{ij}^{*}+\delta_{ij}p$$

For RC structures, the principal structure equation for RC is used to calculate the skeletal stress σ^*^_ij_.

After cracking, Maekawa et al. [48] considered that the pore pressure within the concrete pores (capillary pores and crack voids) is anisotropic. Therefore, assuming that the pore pressures in the crack voids are perpendicular to the parallel crack planes and referring to ASR damage model [52], the total stress *σ_ij_* can be derived from Equation (4):

(4) $$\sigma_{ij}=\sigma_{ij}^{*}+\delta_{ij}\cdot l_{i}\cdot p$$

where *l_i_* is the element direction vector perpendicular to the plane of the crack.

Figure 8 and Figure 9 illustrate the process of calculating the expansion stress of ASR products during the chemical reaction based on pore units, as well as the assessment of the mechanical state of the concrete structure based on expansion-induced damage in the framework units. More detailed computational parameters and additional information can be found in [50].

### 5.2. Finite Element Model and Analysis Methods

**Figure 10:**
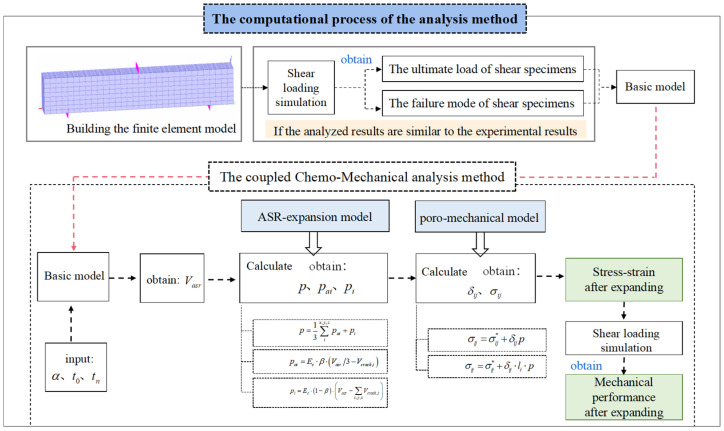
Computational procedure for the simulation method.

The computational process of the analysis method is illustrated in Figure 10. A simulation model for a RC beam was established using the multiscale simulation platform of DuCOM-COM3. By dispersing reinforcement within concrete elements and setting a reinforcement ratio, the model replicates the experimental boundary constraints and loading conditions of the beam. The analysis results are then compared with those obtained from experiments conducted on a control group. If the failure mode matches that observed in experiments and there is good agreement between load–displacement curves, it can be concluded that the analysis model provides accurate results. For the damaged RC beam model, the measured values of ASR gel volume α, ASR initiation time *t_0_*, and ASR termination time *t_n_* are used as inputs. Through calculations, the expansion rate of concrete *ε_t_* is obtained. If the computed concrete expansion rate *ε_t_* matches experimentally measured values, it is considered as an initial condition for mechanical performance analysis by solving Equations (3) to (4). This allows for obtaining load–displacement curves and stress–strain variations in RC beams damaged by ASR. For large-scale concrete structures and diverse engineering environments, the model can further incorporate information regarding the form of the concrete structure and environmental conditions such as temperature, humidity, and alkali content to achieve quantification of expansion rates and predict mechanical properties.

**Figure 11:**
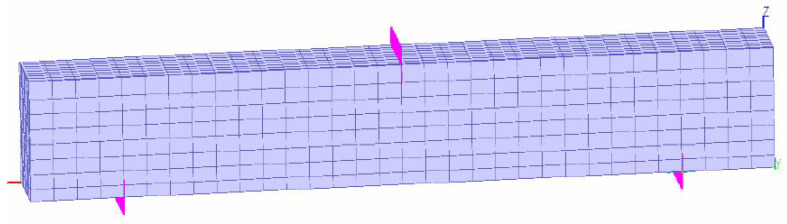
Finite element model of reinforced concrete beam.

The geometric dimensions, reinforcement arrangement, and boundary conditions of the finite element model of the RC beam were consistent with the experimental setup. The overall dimensions of the model are 200 mm × 200 mm × 150 mm, with a clear span of 1000 mm. The tensile reinforcement consists of two 14 mm diameter HRB deformed steel bars, with a cover thickness of 50 mm. The density of the concrete is 2400 kg/m³, and based on the material properties obtained from the experiments, the concrete’s compressive strength is set to 49.57 MPa, tensile strength to 4.9 MPa, elastic modulus to 33,100 MPa, and Poisson’s ratio to 0.22. The elastic modulus of the reinforcement is set to 200,000 MPa, with a yield strength of 621 MPa, Poisson’s ratio of 0.3, and a density of 7800 kg/m³. The RC beam model, shown in Figure 11, is constrained at the bottom support approximately 125 mm from each end, and the load is applied at the mid-span of the beam using displacement-controlled loading.

### 5.3. Results of ASR Expansion

**Figure 12:**
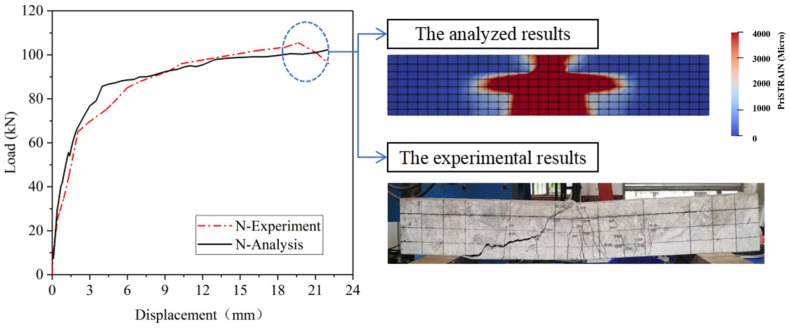
Analytical results of pullout specimens with no ASR damage.

The shear behavior of experimental specimens was simulated using fundamental models and analysis methods, while the computational results were validated through comparison with experimental findings. As shown in Figure 12, the coupled chemical-mechanical analysis approach employed in this study provides a model that accurately simulates the experimental results. The consistency of indicators such as the ascending segment of the load–displacement curve, peak load, and failure mode substantiates this claim. This study focuses on the time of crack appearance, the damage pattern of the beam, and the ultimate load. Although the symmetry of the simulated cracks is not consistent with the test results, both exhibit shear-compression damage in terms of damage pattern. In actual experiments, the occurrence of cracks is random, whereas, in model calculations, the crack simulation assumes an ideally symmetric occurrence. Therefore, the results of this simulation are reliable.

In the intact beam model, the measured ASR gel volume *α*, ASR onset time *t*_0_, and ASR onset end time *t_n_* are utilized as inputs to calculate and solve the ASR expansion model, obtaining the expansion of each component in every beam after ASR damage. As depicted in Equation (5), the expansion rate for each component is determined by integrating the expansion curve within layers based on the finite element model mesh setup. By comparing the experimental and calculated results (as shown in Table 8), it can be observed that this model effectively reproduces site-specific expansions induced by ASR. To further investigate how different levels of ASR damage affect the mechanical properties of RC structures, simulations of RC beam shear damage tests will be conducted based on various states of ASR damage.

(5) $$\beta =\frac{\int_{h2}^{h1}\varepsilon_{t}}{h_{2}-h_{1}}$$

where *h*_1_ indicates the height at the start position of the layer, *h*_2_ represents the height at the end position of the layer, and *ε_t_* signifies the expansion rate on the expansion curve.

### 5.4. Results of Shear Load Simulation

**Figure 13:**
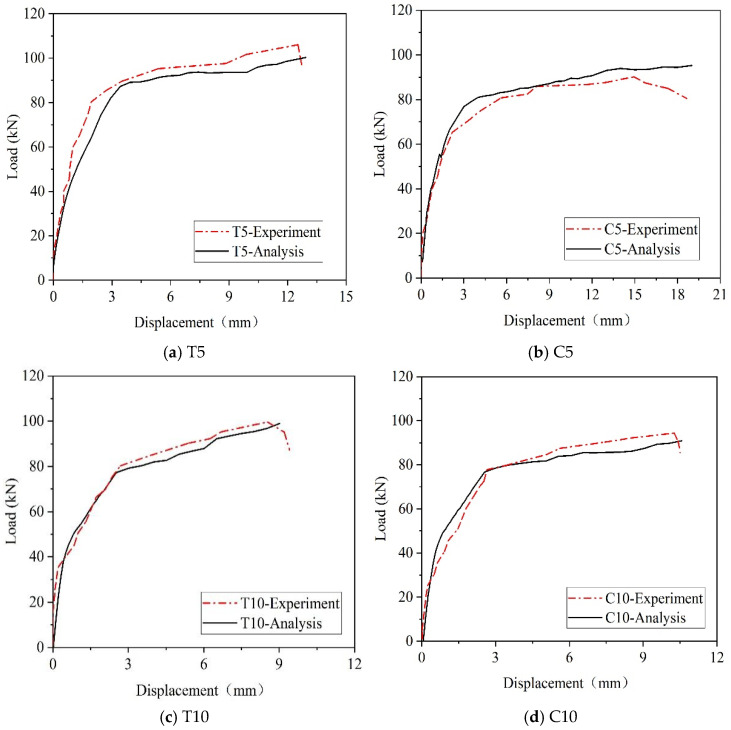
Comparing the experimental results with the analyzed results.

The load–displacement curves in Figure 13 are compared with the corresponding test results, demonstrating a general agreement between the simulation and experimental data. Prior to concrete cracking, the two curves exhibited significant overlap, while after cracking occurred, consistent trends were observed. Therefore, the ascending segment and the peak load of the load–displacement curve can be accurately simulated during the shear process of RC beams. These findings indicate a satisfactory match between the simulation and test results, thereby validating the accuracy of our model.

Table 9 presents a comparison between the cracking load, yielding load, and ultimate load obtained from both analysis and experiments. The results indicate a general agreement between the cracking loads, yielding loads, and ultimate loads of the analysis and experimental data. Hence, it can be concluded that the analytical method effectively replicates the increase in cracking load caused by ASR expansion-induced expansion stresses, as well as the pre-compression stresses generated in concrete due to these expansion stresses. In addition, the analytical method used in this study effectively simulates the interaction between the concrete skeleton and the ASR gel within the pores. The expansion stress from the ASR gel in the pores induces microcracks and other damage within the concrete, while the skeleton units compute the current mechanical state of the concrete structure based on the expansion-induced damage. This approach ultimately allows for the simulation of the deterioration of the mechanical properties of concrete under ASR-induced damage.

**Figure 14:**
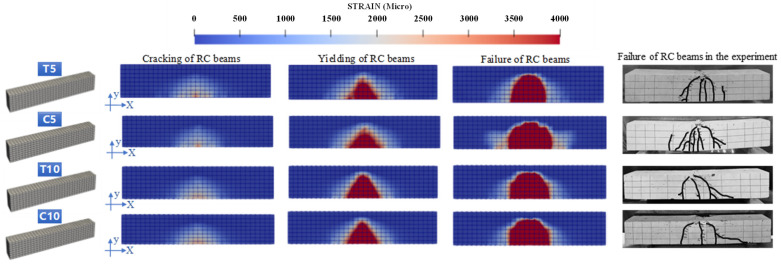
Comparison between simulation analysis results and experimental results of beam failure mode.

The strain curves corresponding to the cracking load, yield load, and ultimate load for RC beams with varying degrees of ASR damage in shear tests are shown in Figure 14. A comparison between Figure 6 and Figure 14 reveals that the simulated damage patterns of the RC beams align well with the experimental results. All four RC beams subjected to ASR damage exhibited the following phenomena: During the preloading phase, tensile strains were primarily concentrated in the lower part of the concrete. Once these strains exceeded the concrete’s ultimate strain capacity, cracks initiated in the tensile zone along the span of the RC beam, leading to overall structural failure. As the load continued to increase, the cracks propagated upward, forming new cracks in the tensile region, and the crack distribution area expanded. Ultimately, failure in the compressive zone resulted in the complete failure of the test beams. However, a comparative analysis of the four beams shows that, due to the use of different accelerated ASR exposure methods, each beam experienced different initial expansion damage. Consequently, the strain distribution at failure varied among the beams.

## 6. Conclusions and Outlooks

### 6.1. Conclusions

This study investigated the expansion mechanism and deterioration characteristics of RC beams affected by ASR damage in various locations. A coupled chemical-mechanical analysis method was proposed to accurately simulate the macroscopic mechanical properties of RC beams. When compared with experimental results, this approach demonstrates a more accurate simulation of the macroscopic mechanical properties under non-uniformly accelerated ASR expansion. Consequently, the conclusions can be drawn as follows:

1. The immersion location and depth exert a significant impact on concrete expansion, with greater expansion occurring in the soaked region compared to the dry area. Cracks tend to concentrate around the immersed region, while reinforcement helps inhibit cracking on tension zone surfaces and compression zone surfaces due to confinement effects.
2. ASR induces an increase in cracking loads of RC beams, particularly pronounced in beams located within the soaked tensile zone. This is attributed to the generation of swelling stresses within the concrete due to ASR, which results in a pre-tensioning stress on the reinforcement. Consequently, this pre-existing stress needs to be counteracted prior to shear testing for crack initiation, thereby leading to an observed increase in cracking loads.
3. Due to the occurrence of ASR-induced micro-cracks in the interface zone between concrete aggregate and cement paste, the strength of concrete is adversely affected, resulting in a reduced load-carrying capacity of RC beams after ASR damage, which is particularly evident in the soaked compression zone.
4. The ASR damage has an adverse effect on the bond between the aggregate and cement paste, resulting in a reduction in beam ductility. Furthermore, for specimens with greater soaking depths, the detrimental impact of ASR damage on ductility becomes more pronounced.
5. The accuracy of the proposed coupled chemical-mechanical analysis method was validated through a comparative analysis between experimental and analytical results. This method has significant potential for future evaluations of the mechanical properties of concrete structures affected by ASR damage.

### 6.2. Outlooks

This study investigates the expansion mechanisms and mechanical performance degradation characteristics of RC beams with ASR damage through mechanical experiments. The research explores the restraining effect of the reinforcement on the transport and expansion of ASR products. A chemical-mechanical damage calculation method for RC considering ASR damage is proposed, and the model’s accuracy is validated through a comparison of experimental results and computed outcomes. By applying the ASR model in engineering construction, combined with the water environment, proportion, aggregate properties, and other data, the concrete alkali aggregate damage in different parts of the structure is predicted. Based on the model results, reinforcing measures can be taken in advance for areas possibly susceptible to serious damage. At the same time, the model can predict the residual bearing capacity of the RC structure with the damage. Based on the model’s long-term prediction results, this is of great significance for the operation and maintenance of the project.

However, the study lacks in-depth analysis regarding the variation in crack width and depth under non-uniform expansion, as well as the changes in the stress–strain behavior of the reinforcement during shear tests. Future research should focus on a more detailed investigation of crack distribution patterns and the strain variation in reinforcement during the loading process.

## Figures and Tables

**Table 1:** Chemical properties of Ordinary Portland cement.

Chemical Composition	CaO (%)	SiO_2_ (%)	Al_2_O_3_ (%)	CO_2_ (%)	MgO (%)	SO_3_ (%)	Fe_2_O_3_ (%)	K_2_O (%)	P_2_O_5_ (%)	TiO_2_ (%)	Total Alkali Content (%)
PO 42.5	66.56	18.5	3.55	0	1.26	3.13	4.03	1.35	0.3	0.6	0.70

Note: The total alkali content of cement is determined by flame photometry.

**Table 2:** Concrete mixture proportions.

Water-To-Cement Ratio	Constituent (kg/m^3^)					Cube Compressive Strength (MPa)
	Cement	Water	Fine Aggregate	Coarse Aggregate	Superplasticizer	
0.45	440	200	443	1158	1.5	49.57

Note: The cube compressive strength is tested using concrete specimens with dimensions of 150 mm × 150 mm × 150 mm.

**Table 3:** List of specimens and experiment variable.

Name	Steel Reinforcement	Reinforcement Ratio(%)	Deterioration Surface	Deterioration Depth (mm)
N	2Φ14	0.77	/	/
T5	2Φ14	0.77	Tension side	50
C5	2Φ14	0.77	Compression side	50
T10	2Φ14	0.77	Tension side	100
C10	2Φ14	0.77	Compression side	100

**Table 4:** Relationship between the cracking load and immerse depth.

Location of ASR Damage	Ratio of the Immerse Depth to the Beam Height		
	0	0.25	0.5
Tension side	29 kN	32.5 kN	37.5 kN
Compression side		30 kN	30 kN

(Note: “Ratio of the immerse depth to the beam height” represents the ratio of the immerse depth to the beam height. The ratio of C5 and T5 is 0.25, and the ratio of C10 and T10 is 0.5).

**Table 5:** Relationship between the maximum load and damage depth.

Location of ASR Damage	Ratio of the Damage Depth to the Beam Height		
	0	0.25	0.5
Tension side	105.5 kN	106 kN	99.6 kN
Compression side		90 kN	94 kN

**Table 6:** Relationship between the ductility coefficient and damage depth.

Location of ASR Damage	Ratio of the Damage Depth to the Beam Height		
	0	0.25	0.5
Tension side	6.91	4.72	3.17
Compression side	6.91	5.33	3.87

**Table 7:** Experimental results.

Name	Deterioration Position	Deterioration Depth (cm)	*P_cr_* (kN)	*P_y_* (kN)	*P_u_* (kN)	*P_cr_*/*P_u_*	Reinforcement Yield	Failure Mode
N	/	/	29	69	105.5	0.27	Yes	Shear compression
T5	Tension side	5	32.5	80	106.0	0.31	Yes	Shear compression
C5	Compression side	5	30	70	90.0	0.33	Yes	Shear compression
T10	Tension side	10	37.5	87	99.6	0.38	Yes	Shear compression
C10	Compression side	10	30	75	94.0	0.32	Yes	Shear compression

**Table 8:** Comparing the experimental expansion rate with the analyzed results.

Specimens		Layer Number of the Cell							
		1	2	3	4	5	6	7	8
T5	α	0.00069	0.00067	0.00061	0.00058	0.00056	0.00054	0.00051	0.00047
	Measured expansion (%)	0.127	0.117	0.108	0.101	0.097	0.094	0.087	0.082
	Calculated expansion (%)	0.125	0.12	0.109	0.104	0.1	0.097	0.091	0.085
C5	α	0.00033	0.00041	0.00045	0.00046	0.00060	0.00064	0.00085	0.00097
	Measured expansion (%)	0.055	0.070	0.077	0.086	0.100	0.119	0.145	0.169
	Calculated expansion (%)	0.06	0.073	0.081	0.082	0.108	0.115	0.153	0.175
T10	α	0.00079	0.00077	0.00073	0.00070	0.00069	0.00066	0.00060	0.00058
	Measured expansion (%)	0.141	0.136	0.133	0.128	0.122	0.116	0.111	0.103
	Calculatedexpansion (%)	0.143	0.138	0.132	0.126	0.125	0.118	0.108	0.105
C10	α	0.00042	0.00046	0.00048	0.00061	0.00074	0.00084	0.00091	0.00093
	Measured expansion (%)	0.072	0.08	0.09	0.105	0.128	0.147	0.159	0.173
	Calculated expansion (%)	0.075	0.082	0.087	0.109	0.134	0.152	0.163	0.168

**Table 9:** Calculated load compared with the measured loads.

Specimens	Cracking Load (kN)			Yielding Load (kN)			Ultimate Load (kN)		
	Calculated Load	Measured Load	Calculated/Measured	Calculated Load	Measured Load	Calculated/ Measured	Calculated Load	Measured Load	Calculated/ Measured
N	27.00	29.00	0.931	66.5	69	0.964	102.30	105.50	0.970
T5	30.00	32.50	0.923	67	80	0.838	100.00	106.00	0.943
C5	28.00	30.00	0.933	79	70	1.129	95.00	90.00	1.055
T10	40.00	37.50	1.067	84.5	87	0.971	98.00	99.60	0.984
C10	32.00	30.00	1.067	75.8	75	1.011	91.00	94.00	0.968
Mean			0.984			0.983			0.995
SD			0.068			0.093			0.039
COV			0.069			0.095			0.040

## Data Availability

All data has been shown in this manuscript.
